# Genotype Analysis of *Bacillus anthracis* Strains Circulating in Bangladesh

**DOI:** 10.1371/journal.pone.0153548

**Published:** 2016-04-15

**Authors:** Farzana Islam Rume, Alessia Affuso, Luigina Serrecchia, Valeria Rondinone, Viviana Manzulli, Emanuele Campese, Pietro Di Taranto, Paritosh Kumar Biswas, Chowdhury Rafiqul Ahsan, Mahmuda Yasmin, Antonio Fasanella, Martin Hugh-Jones

**Affiliations:** 1 Istituto Zooprofilattico Sperimentale of Puglia and Basilicata, Anthrax Reference Institute of Italy, Foggia, Italy; 2 Department of Microbiology, Dhaka University. Dhaka, Bangladesh; 3 Department of Microbiology & Veterinary Public Health, Chittagong Veterinary and Animal Sciences University, Chittagong, Bangladesh; 4 Department of Microbiology & Virology, Patuakhali Science and Technology University, Patuakhali, Bangladesh; 5 School of the Coast & Environment, Louisiana State University, Baton Rouge, United States of America; Naval Research Laboratory, UNITED STATES

## Abstract

In Bangladesh, anthrax, caused by the bacterium *Bacillus anthracis*, is considered an endemic disease affecting ruminants with sporadic zoonotic occurrences in humans. Due to the lack of knowledge about risks from an incorrect removal of infected carcasses, the disease is not properly monitored, and because of the socio-economic conditions, the situation is under-reported and under-diagnosed. For sensitive species, anthrax represents a fatal outcome with sudden death and sometimes bleeding from natural orifices. The most common source of infection for ruminants is ingestion of spores during grazing in contaminated pastures or through grass and water contaminated with anthrax spores. Domestic cattle, sheep and goats can also become infected through contaminated bone meal (used as feed) originating from anthrax-infected carcasses. The present investigation was conducted to isolate *B*. *anthracis* organisms from 169 samples (73 soil, 1 tissue, 4 bone and 91 bone meal samples) collected from 12 different districts of Bangladesh. The sampling was carried out from 2012 to 2015. Twelve samples resulted positive for *B*. *anthracis*. Biomolecular analyses were conducted starting from the Canonical Single Nucleotide Polymorphism (CanSNP) to analyze the phylogenetic origin of strains. The analysis of genotype, obtained through the Multiple Locus Variable Number Tandem Repeat Analysis (MLVA) with the analysis of 15 Variable Number Tandem Repeats (VNTR), demonstrated four different genotypes: two of them were previously identified in the district of Sirajganj. The sub-genotyping, conducted with Single Nucleotide Repeats analysis, revealed the presence of eight subgenotypes. The data of the present study concluded that there was no observed correlation between imported cattle feed and anthrax occurrence in Bangladesh and that the remarkable genetic variations of *B*. *anthracis* were found in the soil of numerous outbreaks in this country.

## Introduction

In Bangladesh, anthrax (known as *“Torka”*) is considered an endemic disease. This infectious non-contagious disease is often fatal for human and for the wide range of animal species. Domestic and wild ruminants are considered the most susceptible. The causative agent is *Bacillus anthracis* but due to the lack of knowledge about risks from an incorrect removal of infected carcasses, the disease is not properly monitored and because of the socio-economic conditions, the situation is under-reported and under-diagnosed [1]. It is not unusual that domestic cattle, sheep and goats can become infected through contaminated bone meal (used as feed) originating from anthrax infected carcasses [2]. The capability of *Bacillus anthracis* to form endospores allows this bacterium to accumulate in grass or water, resulting in one of the most common sources of infection for ruminants that ingest spores during grazing in contaminated pastures [3][4].

Vegetative forms sporulate in 48 hours but in presence of CO_2_, there is no sporulation. This occurs in infected carcasses during the putrefaction process. The rate and the degree of sporulation are influenced by the environmental conditions such as temperature, humidity and available water in the microenvironment, pH, O_2_ availability, sunlight and presence of cations [5].

Spores of anthrax bacilli survive better in alkaline soils rich with organic substances and calcium. Within the soil and until favorable conditions occur, *B*. *anthracis* spends much of its existence in the form of spore. Thereafter, the pathogen starts its life cycle.

Nevertheless, nature provides few opportunities to the bacterium for its reproductive cycle; therefore, the development of an extraordinary pathogenicity is the effective strategy to increase the probability of success against the host immune mechanism [6].

The rapid and intense multiplication of vegetative cells within the host leads it directly to death. Though many of the new generations of bacteria are neutralized by the putrefaction process, a part survives and spreads into the soil as spores. This process ensures the standard of environmental density for the continuation of the species.

Thus, the cases of anthrax every year are the result of a natural and ecological equilibrium that seek to promote the maintenance of the bacterium avoiding the extinction. The high concentration of spores within the soil for long period is also allowed for the presence of an outer layer of the endospore, the exosporium. This represents a large surface to anchor the spore in the ground and maintain cluster density. Spores are acquired by grazing (or browsing) and are able to penetrate the hosts through micro-abrasions or across the intestinal mucosa [7]. In humans, *B*. *anthracis* can penetrate into the organism through micro-abrasions or cuts (developing a cutaneous form), via inhalation of the spores (pulmonary form), and through the consumption of infected meat (intestinal form). If not treated with antibiotics, these three forms are potentially fatal. The ability of the spores to live under extreme environmental conditions and remain viable in soil over a long period of time, added to its virulence and to the fact that endospores are very stable in the environment and easy to cultivate, making *B*. *anthracis* one of the most notorious agents to be potentially misused as a biological weapon or tool of bioterrorism [8][9].

The incidence of human infection depends on the control and prevention of the disease in livestock in the area and the safe disposal of the diseased carcasses. More than 600 people have been diagnosed with anthrax in Bangladesh until the year 2011 [10]. Active immunization is the only known method of preventing anthrax in herbivorous animals in areas where the pasture land is already contaminated with spores [11]. Bangladesh soil conditions, together with ambient temperature and rainfall, represent an ideal situation for the spread of *B*. *anthracis*. The persistence of *B*. *anthracis* in Bangladesh soils is related to a lack of knowledge about butchering sick animals, and disposing of butchering wastes and carcasses where animal graze [1]. Therefore anthrax contaminated bone meals are often unknowingly used as feed supplements for healthy animals and can cause outbreaks in areas where the disease is not normally seen. Unfortunately, in Bangladesh and other developing countries, the vaccination of livestock is not widely available because of the limited volume of vaccine produced.

Bangladesh outbreaks indicate that the disease is no longer sporadic but enzootic [10][11][12]. Outbreak reports reveal that the disease is most prevalent in Sirajganj and nearby districts [11][13]. Particularly noteworthy is that in Bangladesh anthrax is likely an under-diagnosed and under-reported disease. Some diagnostic laboratories base their confirmation of *B*. *anthracis* on the microscopic identification with Gram stain and Polychrome methylene blue stain that reveal respectively Gram-positive rods and the capsule. On site anthrax specific antigen test by Immunochromatographic test may be done. From human cutaneous lesions, sputum or blood, specimen culture can confirm the diagnosis. Direct Polymerase Chain Reaction (PCR) tests on clinical specimens are regarded as an acceptable diagnostic procedure [11].

This research could be defined as an active surveillance due to the fact that analyzing environmental samples means acting on the source where the spores could be more gathered. Thus, the purpose of this study is to focus on the causes which could determine the spread of the bacterium becoming an important tool to control the trend of the disease.

In order to encourage an effective fight against infectious disease, international cooperation programs represent the way to prevent the spread of dangerous pathogen agents. This work focuses on the anthrax Bangladesh situation analyzing soil, bone, bone meal samples thanks to a collaboration between the Istituto Zooprofilattico Sperimentale of Puglia and Basilicata (Italy), Chittagong Veterinary and Animal Sciences University (Bangladesh), the University of Dhaka (Bangladesh) and the Louisiana State University (USA).

## Materials and Methods

**Fig 1:**
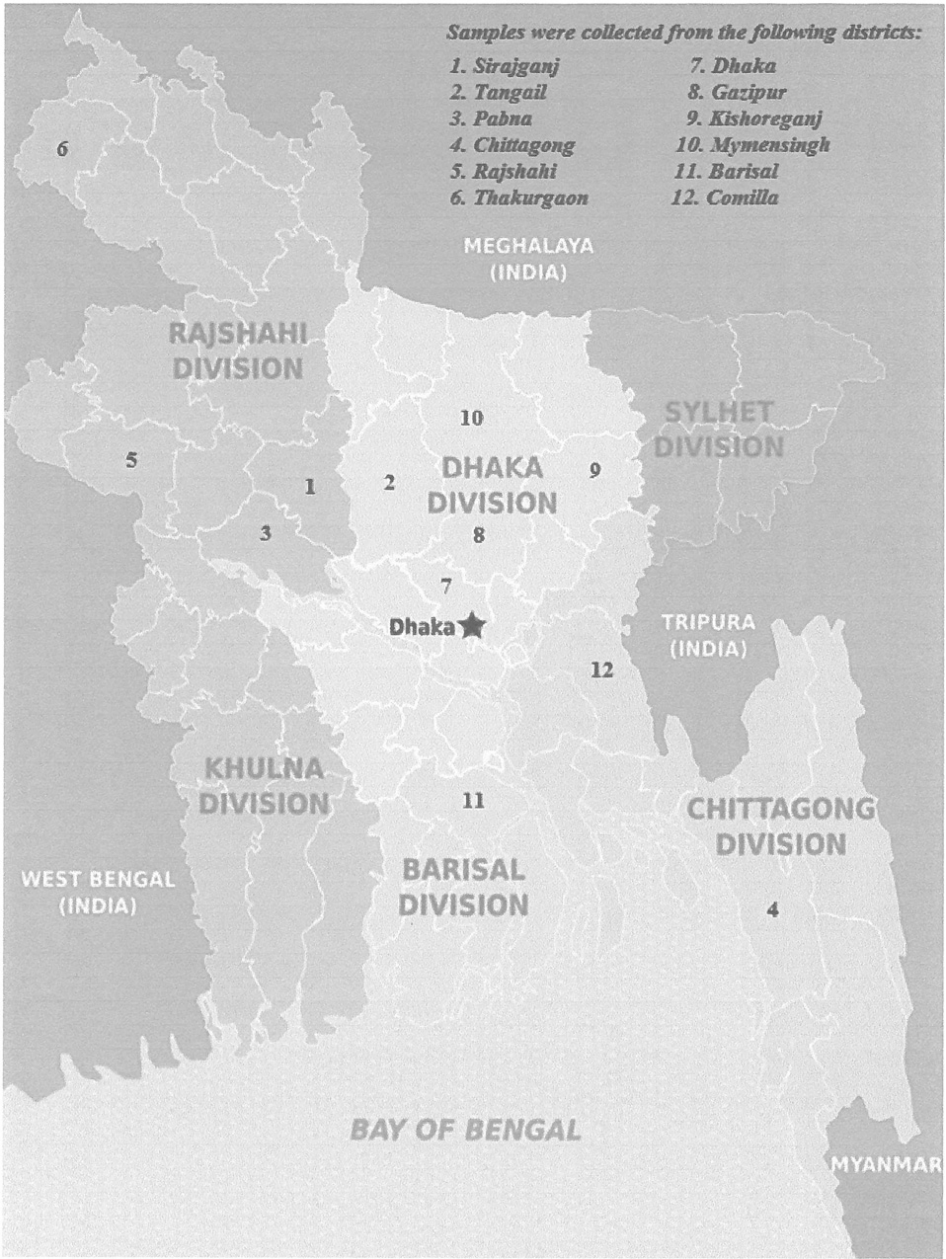
Map of Bangladesh indicating the origin of the samples. *Image modified from “Map of Bangladesh” Author*: *Peter Fitzgerald*, *“World of Maps”- Public Domain (**https*:*//www*.*worldofmaps*.*net/asien/bangladesh/karte-regionen-bangladesch*.*htm**)*

The administration of Bangladesh is divided into eight major regions called divisions (Bengali: Bibhag). The divisions of Bangladesh are divided into 64 districts or zila (Bengali: Jela). The districts are further subdivided into 493 sub-districts or Upazila/Thana (Upojela) [14]. They function as sub-units of districts. A total of 169 samples collected from 12 different districts of Bangladesh: Pabna, Sirajganj, Rajshahi, Comilla, Thakurgaon, Barisal, Chittagong, Dhaka, Gazipur, Kishoreganj, Mymensingh, and Tangail (**Fig 1**), were sent to Foggia. To detect the association between anthrax transmission and imported cattle feed (bone meal), we collected 91 bone meal samples from the cattle feed importer or wholesaler agent from 17 Upazila/Thanas of 10 districts of Bangladesh (Table 1). On the other hand, several outbreak reports reveal that the disease is most prevalent in Sirajganj and nearby districts like Tangail and Pabna [11][13]. Therefore, a total of 73 soil samples were collected from 9 Upazila/Thanas of three districts (Sirajganj, Tangail and Pabna) and randomly picked soil from home stead or burial sites with or without having the history of anthrax cases (Table 1).

**Table 1:** Data on samples and sampling area.

Sl No/Districts	Upazilla/Thana (GPS coordinates)	Village	Soil	Bone	Tissue	Bone meal
1. Sirajganj	Kamarkhanda (24.3667° N to 89.7042° E)	Dhopakandi	6	-	-	-
		Kazipura	1	-	-	-
		Alokdia	1	-	-	-
		Noya chala	1	-	-	-
		Haidarpur	1	-	-	-
		Thakurjipara	1	-	-	-
	Sahajadpur (24.17° N to 89.5883° E)	Gara doho	2	-	-	-
		Binnadae	2	2	1	-
		Nukali	1	-	-	-
		Daya	1	-	-	-
		Char Koujuri	2	1	-	-
		Chithulia	5	-	-	-
		Binotia	4	-	-	-
		Pathaliapara	3	-	-	-
		Mosipur	1	-	-	-
		Jothpar	4	-	-	-
		Jugnidoho	1	-	-	-
		Jamirta	2	-	-	-
		Nogordala	1	-	-	-
		Sarishakoil	1	-	-	-
	Raigonj Upazila, Salanga Union (24.4133°N to 89.5° E)	Raghunathpur	1	-	-	-
		Bhuyagati	1	-	-	-
	Belkuchi (24.2833° N to 89.7166° E)	Adachaki	4	-	-	-
		Somserpur	2	-	-	-
	Ullapara (24.3166° N to 89.5666° E)	Samolipara	1	-	-	-
		Nandigoti	2	-	-	-
		Nobogram	1	-	-	-
		Sonatola	2	-	-	-
2. Tangail	Tangail Sadar (24.2500° N to 89.9167° E)	Katuli	2	-	-	-
	Madhupur (23.9833° N to 89.91666° E)	Ambaria	4	-	-	-
	Dhanbari (24.6166° N to 90.025° E)	North Norilla	5	1	-	-
		Sandalpur	2	-	-	-
		Islampur	2	-	-	-
		Jomserpur	1	-	-	-
3. Pabna	Bera (24.0667° N to 89.6250° E)	South Bongram	2	-	-	-
4. Chittagong	Kotowali (22.3375° N 91.8389° E)	-	-	-	-	32
	Double Mooring (22.3375° N 91.8083° E)	-	-	-	-	4
	Pahartali (22.3667° N to 91.7750° E)	-	-	-	-	2
	Sitakunda (22.3667° N to 91.7750° E)	-	-	-	-	22
5. Rajshahi	Ghoramara (23.9666° N to 89.05° E)	-	-	-	-	2
	Godagari (24.4666° N to 88.3333° E)	-	-	-	-	1
6. Thakurgaon	Pirganj (25.8666° N to 88.3666° E)	-	-	-	-	1
7. Dhaka	Savar (23.8441° N to 90.2511° E)	-	-	-	-	7
	Mirpur (23.5666° N to 90.6166° E)	-	-	-	-	2
	Tejgaon (23.7666° N to 90.4° E)	-	-	-	-	8
8. Gazipur	Gazipur Sadar, Kashimpur union (23.9833° N to 90.3166° E)	-	-	-	-	2
	Kapasia (24.4166° N to 90.0333° E)	-	-	-	-	2
	Sreepur (24.2000° N 90.4667° E)	-	-	-	-	1
9. Kishoreganj	Bajitpur (24.2166° N to 90.95° E)	-	-	-	-	2
10. Mymensingh	Mymensingh Sadar (24.6333°N to 90.1833°E)	-	-	-	-	1
11. Barisal	Barisal Sadar (22.7000° N 90.3667° E)	-	-	-	-	2
12. Comilla	Comilla Sadar (23.4666° N to 91.1833° E)	-	-	-	-	2
**Total (169)**			**73**	**4**	**1**	**91**

The sampling was carried out in the period from 2012 to 2015. The study protocol was reviewed and approved by the faculty of Biological Science, the University of Dhaka (DU), Bangladesh. This study was carried out in strict accordance with the recommendations found in the guide for the use of tissue samples of the DU. The tissue and bone samples were collected from the open fields where the owner threw their dead carcass. The field team obtained informed verbal consent from respondents. No specific permissions were required for the locations from where the environmental samples (bone meal, soil), tissue samples and bone samples of dead animals were collected. Moreover and none of the specimen collections involved either endangered or protected species. No samples were collected from the live animal. Thus, no permits were required from the farm owner. Only verbal consent from farm owners were taken for the collection of soil from the burial sites. Sampling methods were supported by Anthrax in Humans and Animals- 4th edition, as published by the WHO, OIE and FAO. This consent procedure was approved by the faculty of Biological Science, the University of Dhaka (DU), Bangladesh. The whole shipping-process was in accordance with International Bio-Security rules from NC State University in compliance with the US Department of Transportation (DOT) and the International Air Transporters Association (IATA). The biological materials of this study belonged to “Category B infectious substances” that have the proper shipping name “Biological Substance, Category B” and the identification number UN 3373. The samples were properly tripled packaged and compliant with IATA packing instruction.

### Isolation

To detect *B*. *anthracis* from the samples, the Ground Anthrax Bacillus Refined Isolation (GABRI) method was used [15], which had been developed in the Istituto Zooprofilattico Sperimentale of Puglia and Basilicata, Italian Reference Centre of anthrax. This test is able to isolate *B*. *anthracis* in high contaminated soil.

The GABRI method is more sensitive in revealing the presence of *B*. *anthracis* since it involves the use of a non-ionic detergent, such as Tween 20, which allows the separation of spores from soil particles by disrupting hydrophobic interactions with the solid matrices. In addition, the method provides the use of an antibiotic, Fosfomycin that strongly reduces other microbial contaminants.

Each sample has been washed with Washing Buffer (sterile deionized water solution containing 0.5% of Tween 20) and incubated at 64 °C for 20 minutes to eliminate vegetative forms of *B*. *anthracis*. After this incubation, 1 ml of supernatant was mixed with 9 ml of Tryptose Phosphate Broth containing 125 μL/ml of Fosfomycin. From this mix, 500 μL have been seeded on Trimethoprime-Sulfamethoxazole-Polymixine agar plates (TSMP) and incubated, aerobically, at 37°C for 24 hours. Subsequently, suspected colonies (whitish and without hemolysis) were picked and spread onto 5% blood agar and incubated at 37°C for 24 hours. This protocol was used for the processing of all samples received. All tests were run in the Institute’s BL-3 Security Laboratory.

### DNA preparation and Real Time PCR assay

At the same time, a few suspect colonies were scraped off the plate and transferred into 1.5 ml reaction tubes filled with 500 μL of sterile water and subjected to DNA extraction with heat through the use of a thermoblock (98°C for 20 minutes), followed by centrifugation at 12851 relative centrifugal force (rcf) for 10 minutes at 4°C. Specific PCR assays were used to confirm *B*. *anthracis*. The method is based on the amplification of DNA specific sequences through the use of three pairs of specific primers [16].

- The R1/R2 primers are specific for the BA813 gene, located on the chromosome of *B*. *anthracis*;
- PAG 23/24 primers are specific for the protective antigen (PA), located on the pXO1 virulence plasmid;
- CAP 57/58 primers are specific for the capsule, plasmid pXO2.

The amplification was performed using the CFX Connect Real Time PCR Detection System. The Melting curve was determined by increments of 0.5°C starting from a temperature of 65°C to 95°C and was analyzed by CFX Manager^TM^ Software, Version 3.0.

For the following biomolecular analysis, high quality DNA is required. Thus, colonies which had tested positive to the RT-PCR assay and previously seeded on 5% sheep blood agar, were selected for DNA extraction using the DNAeasy Blood and Tissue kits (Qiagen, USA) following the protocol for Gram-positive bacteria.

### PHRANA (Progressive Hierarchical Resolving Assays using Nucleic Acids)

In this study to investigate the diversity of *B*. *anthracis* isolates, the hierarchical fingerprinting system PHRANA was applied. Thus, canSNP analysis was conducted as a preliminary test to confirm the origin of strains. Subsequently, Multiple Loci Variable Number Tandem Repeat Analyses (MLVA) were performed which exhibits greater resolving power than canSNP analysis and allows differentiation between closely related strains of the same canSNP group. For a finer genotype resolution, isolated strains were subjected to SNR analyses, which can show a high rate of mutation (6.0 x 10^−4^ mutations per generation) and allows verification of SNR minimal genetic differences within the same genotype.

### Canonical Single Nucleotide Polymorphism analysis

CanSNP assay is a phylogenetic approach to identify SNPs that efficiently partition bacterial strains into genetic groups consistent with their recognized population structure. Following the method described by Van Ert et al., we were able to reveal the group of strains isolated from our samples and detect to which conserved group or lineage they belonged. Phylogenetic analyses, as well as information on isolate frequencies and global geographic distribution, facilitate the overview on the global diversity and historical transmission patterns of this pathogen [17].

Since this test subdivides all of the *B*. *anthracis* isolates into three previously recognized major lineages (A, B and C), with further subdivisions into one of 12 distinct sub-lineages or sub-groups, each DNA extracted from our samples that had positive to PCR analysis, was processed for CanSNP identification using 13 TaqMan-Minor Groove Binding (MGB) allelic discrimination assays with oligonucleotides and probes as described by Van Ert et al., for each of the 13 canonical SNPs. The results obtained by CanSNPs were compared to the recognized 12 sub-lineage or sub-groups [17].

### Multiple-Locus Variable Number Tandem Repeat with 15 VNTRs

Molecular typing of *B*. *anthracis* strains is based on MLVA. The method is based on two approaches described firstly by Keim et al. [18] (who identified 8 loci to genotype *B*. *anthracis*) and subsequently compiled together with the addition of 7 new loci, into a multiple-locus VNTR analysis with the final protocol described by Van Ert et al., who identified the genotyping of *B*. *anthracis* [17]. All the VNTRs markers provide high levels of discrimination among different isolates also monitoring for the presence or absence of the plasmids as well as for plasmid-based variation. All isolates were subjected to 15-loci MLVA analysis using *B*. *anthracis* reference strain Ba001/1FG previously analyzed by Van Ert et al. [17] with designation number A0280ITA and then by Keim et al. [18] with designation number K0021.

Seven PCR reactions amplifies fifteen VNTRs loci present at the level of the chromosome (vrrA, vrrB1, CG3, vrrB2, vntr19, vrrC1, vrrC2, m vntr32, vntr12, vntr35, vntr23) and at the level of plasmids (vntr16, vntr17, pxO1, pxO2). MLVA test provided the preparation of two Singleplex and five Multiplex reactions, in a final volume of 15 μL.

The MLVA PCR products were diluted 1:80 and subjected to capillary electrophoresis on an ABI Prism 3130 genetic analyzer (Applied Biosistems Inc.) with 0.25μL GeneScan 1200, and sized by Gene Mapper 4.0 (Applied Biosistems Inc.).

### Single Nucleotide Repeats (SNR)

To obtain deeper information about the genetic diversity within a single outbreak, Single Nucleotide Repeats analysis gives a fine scale of resolution among isolates belonging to the same genotype. Single nucleotide repeats, also named mononucleotide-nucleotide repeats, are a type of VNTR (Variable Number Tandem Repeat) with a high rate of mutation (6.0 x 10^−4^ mutations per generation) present within the genome of *B*. *anthracis*.

In this study, we applied the modified SNR technique described by Kenefic and associates [19]. To identify, four SNRs were set up two different kind of reactions mixture in a final volume of 12.5 μL. Amplified SNR PCR products were diluted 1:80 and subjected to capillary electrophoresis on ABI Prism 3130 Genetic Analyzer (Applied Biosistems Inc.) with 0.25 μL GeneScan 120 LIZ and sized by Gene Mapper 4.0 (Applied Biosistems Inc.).

## Results

Among the 169 samples tested, 12 were positive to RT-PCR analysis: 10 soil and 2 bone samples. For each positive sample few colonies for PHRANA Assay were chosen randomly.

### Canonical Single Nucleotide Polymorphism (CanSNP)

CanSNP analyses for the phylogenetic clustering showed that all isolates belonged to the lineage A major subgroup A.Br. 001/002, which represent the same group circulating in China and other countries in South-East Asia. Furthermore, the A lineage isolates are widely distributed and are found in all over the world; probably this is due to the fact that the A lineage genotype have a better fitness and adaptability [17].

### Multiple- Locus Variable Number Tandem Repeat with 15 VNTRs

**Fig 2:**
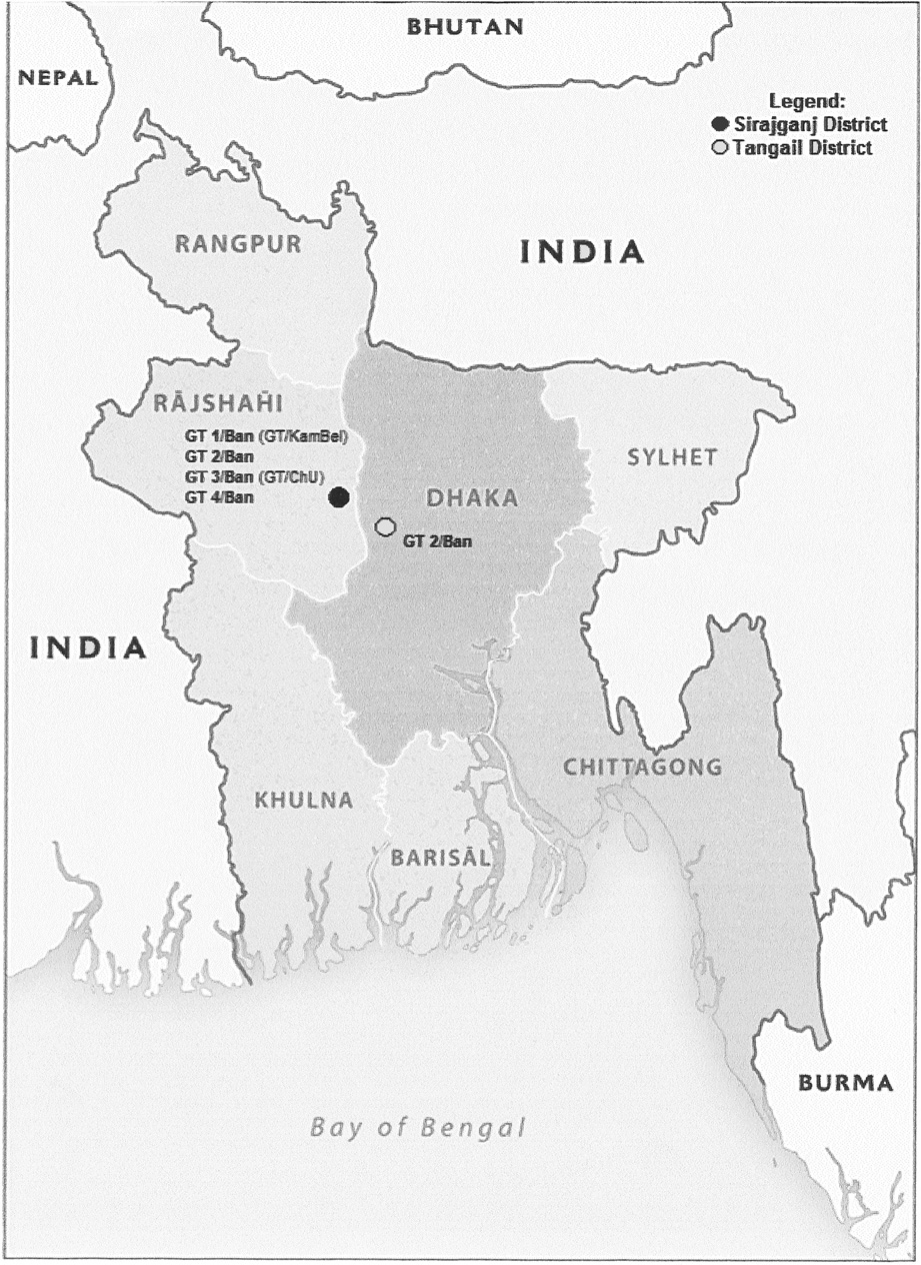
Map of Bangladesh indicating the genotype distribution of *B*. *anthracis*. *Image modified from “Bangladesh Administrative-2011”*, *803502AI (G00535) 6–11*. *Maps at the CIA—Public Domain (**https*:*//www*.*cia*.*gov/library/publications/resources/cia-maps-publications/index*.*html**)*.

The MLVA-15 loci analysis of the isolates demonstrated the presence of four genotypes that were named GT1/Ban, GT2/Ban, GT3/Ban, GT4/Ban, in the district of Sirajganj and genotype GT2/Ban also present in the district of Tangail (**Table 2**) (**Fig 2**). The data reported in this study confirmed the presence of two genotypes (GT1/Ban and GT3/Ban, which corresponded respectively to genotypes GT/KamBel and GT/ChU) already identified from a previous study conducted in the district of Sirajganj and had also shown the existence of two new genotypes, named as GT2/Ban and GT4/Ban, not previously encountered [10].

**Table 2:** MLVA 15- VNTRs results.

		VNTRs observed (bp)																
District	No. Isolates	vrra	vrrb1	vrrb2	vrrc1	vrrc2	cg3	pXO1	pXO2	vntr12	vntr16	vntr17	vntr19	vntr23	vntr32	vntr35	MLVA 15 GT	Fasanella et al. [10] GT
Sirajganj	13	**306**	223	154	584	522	153	**127**	133	110	135	381	90	182	**378**	116	**GT 1/Ban**	GT/KamBel
Sirajganj Tangail	5 18	**306**	223	154	584	522	153	**121**	133	110	135	381	90	182	**378**	116	**GT 2/Ban**	**NEW**
Sirajganj	14	**295**	223	154	584	522	153	**127**	133	110	135	381	90	182	**563**	116	**GT 3/Ban**	GT/ChU
Sirajganj	4	**306**	223	154	584	522	153	**127**	133	110	135	381	90	182	**563**	116	**GT 4/Ban**	**NEW**

### Single Nucleotide Repeats

SNR assay highlighted the existence of four subgenotypes within the GT1/Ban, two subgenotypes within the GT3/Ban, while the GT2/Ban and GT4/Ban showed both one subgenotype (**Table 3**).

**Table 3:** SNR results.

					SNR considered			
District	Genotype (GT)	SubGt	Sample	No. Isolates	HM1 CL33/pXO2	HM2 CL10/Chrom	HM6 CL12/Chrom	HM13 CL35/pXO2
Sirajganj	**GT 1/Ban**	subGt1	**17**—soil	9	82	111	90	118
		subGt2	**17**- soil	1	**83**	111	90	118
		subGt3	**61**- soil	2	**84**	111	90	118
		subGt4	**101**- soil	1	82	**110**	90	118
	**GT 2/Ban**	subGt5	**21—**bone	5	82	109	90	119
	**GT 3/Ban**	subGt6	**79**- soil	7	78	107	91	118
			**81**- soil	6	78	107	91	118
		subGt7	**81**- soil	1	78	**106**	91	118
	**GT 4/Ban**	subGt8	**77**—soil	4	82	109	92	119
Tangail	**GT 2/Ban**	subGt5	**87—**bone	5	82	109	90	119
			**87**—soil	3	82	109	90	119
			**91**- soil	1	82	109	90	119
			**93**- soil	7	82	109	90	119
			**95**- soil	2	82	109	90	119

## Discussion

*B*. *anthracis* is considered one of the bacteria with a high degree of genetic homogeneity and this feature makes it difficult to discriminate among the bacterial strains. The phenomenon of high genetic homogeneity is motivated by the high spore survival capacity which has allowed *B*. *anthracis* to multiply a relatively limited number of times during its evolution.

The present research was conducted analyzing 169 samples (73 soil, 1 tissue, 4 bone and 91 bone meal samples) collected from 12 different districts of Bangladesh. The sampling was carried out from 2012 to 2015. This study reveals to be an important tool for the active surveillance based on the monitoring of suspected anthrax foci.

For this reason, it was important to collect different kind of samples in order to inspect the reason of anthrax transmission, control the spread of the disease, and consequentially reduce the phenomenon. Thus, a total of 91 bone meal samples were collected from the cattle feed importer or wholesaler agent from 17 Upazila/Thanas of 10 districts of Bangladesh (Table 1) to detect the association between anthrax transmission and imported cattle feed (bone meal). Furthermore, as several outbreak reports reveal that the disease is most prevalent in Sirajganj and nearby districts like Tangail and Pabna [11][13] a total of 73 soil samples were collected from 9 Upazila/Thanas of these three districts, randomly picking soil from homestead or burial sites with or without having the history of anthrax cases (Table 1). All of the 91 bone meal samples were negative and the results might nullify the hypothesis of anthrax transmission through imported cattle feed. On the other hand, 10 soil samples were positive out of 73. Here, each soil sample represented individual farm of the village with or without having the previous history of anthrax outbreak. After the isolation of *B*.*anthracis*, biomolecular assays allowed to find out the genetic diversity among the isolates of same and different samples and to depict the persistence of the organism in the environmental samples rather than the calculation of prevalence of the disease.

Using the CanSNP analysis, it has been possible understand the phylogenetic origin of isolates and confirm that all the strains analyzed in this study, belonged to A.Br. 001/002, typical of strains circulating in China and other countries in South-East Asian.

MLVA is a standard tool for *B*. *anthracis* genotyping.This molecular technique has proved to be useful for molecular typing of *B*. *anthracis* is the analysis of VNTR sequences. These are short nucleotide sequences, tandemly repeated and in a variable number of copies which give rise to length polymorphisms easily detectable by the PCR technique. The several loci analyzed, such as hypervariable regions, can be used for discrimination between different strains. The analysis of these hypervariable regions with methods such as MLVA is a valuable tool for studying the diversity, evolution and molecular epidemiology of *B*. *anthracis*. Because of the high homoplasy of VNTR loci, MLVA utility is limited by the difficulties in understanding how these genotypes are related to each other. Nevertheless, MLVA represents a valid method for gaining an overall view on the genetic diversity of *B*. *anthracis* within a country. The MLVA analysis of the isolates revealed the presence of four genotypes: GT1/Ban, GT2/Ban, GT3/Ban and GT4/Ban. All the samples with these genotypes came from Sirajganj district, in Nothern Bangladesh. In particular, GT/2 Ban has been found also in Tangail district, geographically very close to Sirajganj. Gt1/Ban differed from GT2/Ban, in the size of pXO1, from GT3/Ban in relation to vrra and vntr32 and from GT4/Ban for the variation in locus vntr32. GT1/Ban was already identified in a previous study by Fasanella et al. [10] and it was named as GT/KamBel, that took the name from the area from which they were isolated: Kamarkhand and Belkuchi. GT3/Ban was also identified in the same study and called GT/ChU because the genotype was the same in Chitulia village and Ullapara subdistrict. GT2/Ban and GT4/Ban resulted as new genotypes, earlier undiscovered. (**Table 2**) (**Fig 2**)

For what concerns the SNR analysis, it has been demonstrated a high discriminatory power among all the isolates with 8 SNR types detected. These findings confirm the ability of SNRs to mutate rapidly, suggesting the presence of mutational step during the multiple replications. In our study, this hypothesis is strengthened by presence of two different sub-genotypes in the same sample: subGt1 e subGt2 identified within the GT1/Ban deriving from a unique soil sample. This occurs also for subGt6 and subGt7 within GT3/Ban, discovered in isolates coming from a distinct soil sample. (**Table 3**)

Thus, in this context, the SNR analysis detecting sub-genotypes, allows one to understand how many variations are in the same genotype, paving the way to further investigations that might explain which other causes intervene in the genetic variations of *B*. *anthracis*. The presence of genetic variations identified using MLVA and SNR analysis, allows us to understand that the diversity within the A.Br. 001/002 group arises from the evolution of a strain ecologically established and not recently introduced.

In the present study the high percentage of positive soil samples allows us to consider soil as one of the major source for the spread of *B*. *anthracis* and for the animal infection. The practice of burial of dead animals and the improper removal of infected carcasses are the mainstay of causes that determine the contamination and persistence of bacteria in the environment.

The negative results of bone meal samples probably nullify the hypothesis of anthrax transmission through the imported cattle feed supplement; may be a good news for the cattle feeds trades of Bangladesh.
